# Endovascular coiling versus microsurgical clipping for ruptured intracranial aneurysms: a meta-analysis and systematic review

**DOI:** 10.1186/s41016-022-00283-3

**Published:** 2022-07-25

**Authors:** Chao Peng, Yu-hang Diao, Shi-fei Cai, Xin-yu Yang

**Affiliations:** Department of Neurosurgery, Tianjin Medical University General Hospital, Tianjin, The People’s Republic of China

**Keywords:** Ruptured intracranial aneurysms, Coiling, Clipping, Meta-analysis

## Abstract

**Background:**

The purpose of this analysis is to evaluate the current evidence with regard to the effectiveness and safety between coiling and clipping in patients with ruptured intracranial aneurysms (RIAs).

**Methods:**

We performed a meta-analysis that compared clipping with coiling between July 2000 and September 2021. PubMed, EMBASE, and the Cochrane Library were searched for related articles systematically. And the treatment efficacy and postoperative complications were analyzed.

**Results:**

We identified three randomized controlled trials and thirty-seven observational studies involving 60,875 patients with ruptured cerebral aneurysms. The summary results showed that coiling was related a better quality of life (mRS0-2; OR=1.327; CI=1.093–1.612; *p*<0.05), a higher risk of mortality (OR=1.116; CI=1.054–1.180; *p*<0.05), higher rate of rebleeding (RR=1.410; CI=1.092–1.822; *p*<0.05), lower incidence of vasospasm (OR=0.787; CI=0.649–0.954; *p*<0.05), higher risk of hydrocephalous (RR=1.143; CI=1.043–1.252; *p*<0.05), lower risk of cerebral infarction (RR=0.669; CI=0.596–0.751; *p*<0.05), lower risk of neuro deficits (RR=0.720; CI=0.582-0.892; *p*<0.05), and a lower rate of complete occlusion (OR=0.495; CI=0.280-0.876; *p*<0.05).

**Conclusion:**

Coiling was significantly associated with a better life quality (mRS0-2), a lower incidence of postoperative complications, and a higher rate of mortality, rebleeding, hydrocephalous, and a lower rate of complete occlusion than clipping.

**Supplementary Information:**

The online version contains supplementary material available at 10.1186/s41016-022-00283-3.

## Background

Aneurysmal subarachnoid hemorrhage (aSAH) is accounted for 80% of cases of nontraumatic subarachnoid hemorrhage (SAH) [1], contributing to significant mortality. There are two procedures for the treatment of aSAH: microsurgical clipping and endovascular coiling [2]. The first clipping operation was published by Walter Dandy in 1937 [3]. In 1991, the Guglielmi detachable coil for coiling was found, putting a platinum coil into a cerebral aneurysm [4]. The goal of treatment was to occlude the aneurysm to reduce the risk of bleeding. Given this purpose, clipping and coiling are both effective, although there remain controversial with regard to which treatment strategies are better for patients with aSAH.

Until 2002, the International Subarachnoid Aneurysm Trial (ISAT) demonstrated that individuals who underwent coiling were associated with a less morbidity and mortality at 1-year follow-up compared with clipping [5]. This finding contributed to endovascular coiling had been widely accepted becoming the preferred strategy of treatment at many centers [6]. However, the results of ISAT also caused some criticism, such as 7416 of the 9559 patients with ruptured intracranial aneurysms (RIAs) were excluded, the location, and type of intracranial aneurysms (IAs) as well as types of recruiting centers were widely different, and the proficiencies of the performer of coiling and clipping were varied [5, 7].

In recent years, some randomized controlled trials (RCTs) and retrospective comparative studies and prospective studies have also been published, and some results of these publications were different from ISAT [8]. As a result, there remains some debate about the choice of coiling and clipping for patients with aSAH, while it is the aim of this meta-analysis and systematic review to evaluate the two treatments’ effectiveness and complications from a great deal of evidence containing RCTs and observational studies to provide a guiding strategy in selecting which treatment methods to perform in patients with aSAH.

## Methods

The Preferred Reporting Items for Systematic Reviews and Meta-analyses (PRISMA) [9] was used for this meta-analysis guidelines. And we compared the two treatments by primary outcomes (treatment efficacy) and secondary outcomes (postoperative complications).

### Systematic literature search

We searched all literatures with regard to the comparison between coiling and clipping for ruptured intracranial aneurysms (RIAs) through PubMed, MEDLINE, EMBASE, and Cochrane Library databases systematically and comprehensively. The date of these studies was ranged from 2000 to 2021. The search strategies were conducted using “ruptured intracranial aneurysms,” “coiling,” and “clipping,” as our search terms and keywords. A manual search for literatures that was referenced by other publications but met our inclusion criteria was conducted as a supplement. We would use the most current literature, when a study produced multiple papers.

### Inclusion and exclusion criteria

Literatures were included if they met the PICOS criteria: (1) population: limited the comparison to the RIAs individuals; (2) intervention: used coiling and clipping; (3) comparison: compared the results after coiling and clipping; (4) outcome measures: the results after treatment and the follow-up; and (5) an official published RCTs or non-RCT.

The exclusion criteria were as follows: (1) Letters to the editor and commentary or conference articles and (2) animal trials; (3) unclear patient outcome data; (4) case reports and case series; (5) systematic reviews or meta-analyses; and (6) other types of IAs, such as trauma.

### Selection and data extraction

The data were extracted independently by two observers, C Peng, SF Cai, and YH Diao, containing basic data (author, publication time, age), study characteristics (trial type), and outcomes (rebleeding; mortality; complete occlusion, complications of postoperative) in a table. The senior investigator (YY Yang) would review the data for completeness and accuracy.

### Statistical analyses and quality assessment

The results of this study were analyzed by standard software (Stata version 12.0 statistical software). For categorical variable results, risk ration (RR) or odds ratios (ORs) with 95% confidence intervals (CIs) were tested for result assessment. When *I*^2^>50%, the data was treated as obvious heterogeneity; therefore, a meta-analysis was performed using random effect model. Otherwise, the fixed effect model was conducted. For continuous variable results, standard mean difference (SMD) or weighted mean difference (WMD) with 95% CIs were calculated for assessment. When *I*^2^>50%, the data was treated as obvious heterogeneity, and the data analysis was conducted by a random effect model. Otherwise, the fixed effect model was conducted. The quality of the RCT literatures were assessed by Cochrane Collaboration’s tool, and the Newcastle-Ottawa scale were used to evaluate the quality of the observational studies.

## Result

### Quality of included studies

The article quality assessment was conducted separately by three reviewers, C Peng, YH Diao, and SF Cai. Thirty-seven observational studies were assessed by the Newcastle-Ottawa scale, and the Cochrane Risk of Bias Tool was used to assess the quality of the 3 RCTs. And the results were showed in Table 1 and Additional file 1.

**Table 1:** The Newcastle-Ottawa scale for quality assessment observational studies

**Trials**	**Representativeness cohort**	**Exposure Ascertainment**	**Comparability**	**Outcome Assessment**	**Sufficient Duration**	**Adequacy of follow up of cohorts**
**Kelly et al.**	Yes	database	No restricton, Matched in 1,2,5,6	record linkage	Yes	Yes
**Choi et al.**	Yes	database	Restricted to MCA, Matched in 1,2,5,6	record linkage	Yes	Yes
**Ayling et al.**	Yes	database	No restricton, Matched in 1,2,4,5,6	record linkage	No	Yes
**Berro et al.**	Yes	medical record	Restricted to MCA, Matched in 1,2,4	record linkage	No	Yes
**Darsaut et al.**	Yes	database	No restricton	record linkage	Yes	Yes
**Zanaty et al.**	Yes	database	No restricton, Matched in 1,2,4,5,6	record linkage	No	Yes
**Heit et al.**	Yes	medical record	Restricted to ACOA, Matched in 1,2,3	record linkage	No	Yes
**Scheller et al.**	Yes	medical record	No restricton, Matched in 1,2,3, 6	record linkage	Yes	Yes
**Koh et al.**	Yes	medical record	No restricton, Matched in 1,2,6	record linkage	No	Yes
**Shen et al.**	Yes	medical record	Restricted to Anterior Circulation, Matched in 1,2,4,5	record linkage	No	Yes
**Zhao et al.**	Yes	medical record	No restricton, Matched in 1,2,4,5,6	record linkage	Yes	Yes
**McDonald et al.**	Yes	database	No restricton, Matched in 1,2	record linkage	unclear	unclear
**Li et al.**	Ysa	medical record	No restricton, Matched in 1,2,4,5,6	record linkage	Yes	Yes
**Yu et al.**	Yes	medical record	No restricton, Matched in 1,2,5	record linkage	Yes	Yes
**Bekelis et al.**	Yes	database	No restricton, Matched in 1,2	record linkage	Yes	Yes
**Li et al.**	Yes	medical record	No restricton, Matched in 1,2,3,5,6	record linkage	Yes	Yes
**Deutsch et al.**	Yes	database	No restricton, Matched in 1,2	record linkage	Yes	Yes
**Ryttlefors et al.**	Yes	medical record	Restricted to ≥65 years, Matched in 1,2,4,5,6	record linkage	Yes	NO
**Wadd et al.**	Yes	medical record	Restricted to ACOA, Matched in 1,2,4	record linkage	Yes	Yes
**Hoh et al.**	Yes	database	Restricted to ≥18 years, Matched in 1,2	record linkage	unclear	unclear
**Brunken et al.**	Yes	medical record	No restricton, Matched in 1,2,3,6	record linkage	NO	Yes
**Taweesomboonyat et al.**	Yes	medical record	Restricted to PCOA, Matched in 1,2,3,4,5,6	record linkage	Yes	Yes
**Zhao et al.**	Yes	medical record	Restricted to ACOA, Matched in 1,2,4,5	record linkage	Yes	Yes
**Klompenhouwer et al**	Yes	medical record	No restricton, Matched in 1,2,3,5,6	record linkage	Yes	Yes
**Liao et al.**	Yes	medical record	Restricted to Anterior Circulation, Matched in 1,2,3,4	record linkage	Yes	Yes
**Zhang et al.**	Yes	medical record	Restricted to 60 years, Matched in 1,2,3,6	record linkage	Yes	Yes
**Lusseveld et al.**	Yes	medical record	Restricted to basilar tip aneurysm, Matched in 1,2,4,5	record linkage	No	Yes
**Varelas et al.**	Yes	medical record	No restricton, Matched in 1,2,3,6	record linkage	Yes	Yes
**Hoh et al.**	Yes	medical record	Restricted to age older than 18	record linkage	Unclear	Unclear
**Li et al.**	Yes	medical record	No restricton	record linkage	Yes	Yes
**Liu et al.**	Yes	medical record	No restricton, Matched in 1,2,3,5	record linkage	Yes	Yes
**Gross et al.**	Yes	medical record	No restricton, Matched in 1,2,3,6	record linkage	Unclear	Unclear
**Suzuki et al.**	Yes	medical record	No restricton, Matched in 1,2,4,5,6	record linkage	Yes	Unclear
**Zaidat et al.**	Yes	medical record	No restricton, Matched in 1,2,3,6	record linkage	Unclear	Unclear
**Niskanen et al.**	Yes	medical record	No restricton, Matched in 1,2,3,5,6	record linkage	Yes	Yes
**Rabinstein et al.**	Yes	medical record	No restricton, Matched in 1,2,4,6	record linkage	Yes	Yes
**Kim et al.**	Yes	medical record	Restricted to anterior choroidal artery aneurysms, Matched in 1,2,3,5	record linkage	Yes	Yes

Note: 1 = Age; 2 = Sex; 3 = Hunt and Hess Grade; 4 = World Federation of Neurological Societies Scale; 5 = Aneurism size; 6 =Aneurism location

### Search results and study characteristics

**Fig. 1:**
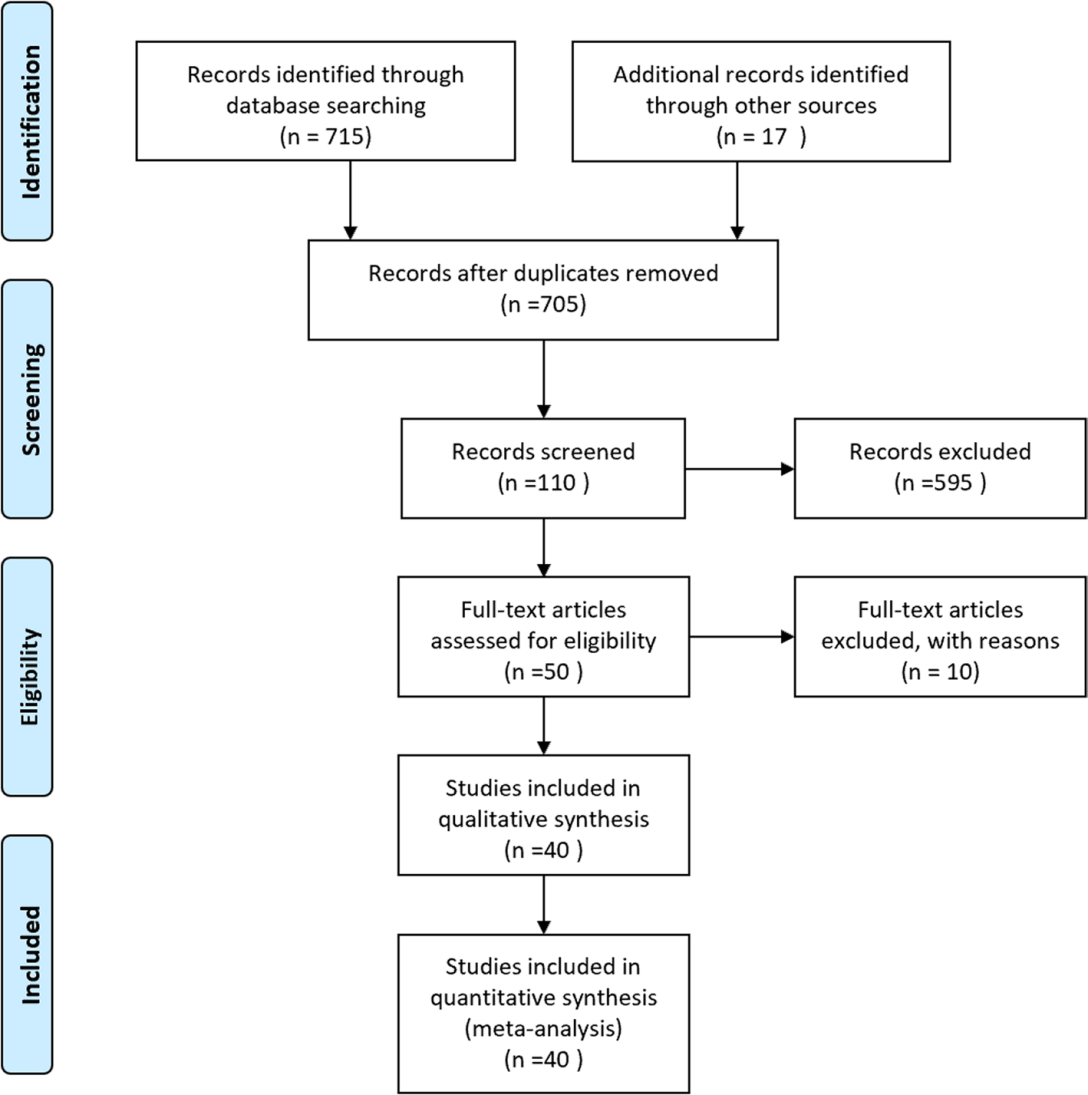
Forty articles met the inclusion criteria and were included in this meta-analysis

Initially, 715 literatures were found by searching an electronic database, and 17 articles were identified by manual search. And there were 705 articles after duplicates were removed. 595 publications were deleted by preliminary screening, ultimately, 40 articles met the inclusion criteria and were included in this meta-analysis. The details were shown in the flow chart (Fig. 1). There were 3 RCTs and 37 observational studies [7, 10-43]. A total of 60,875 patients were included and the size of the sample ranged from 32 to 21,905, 31,791 patients were treated by coiling, 29,084 individuals performed by clipping, and other information was shown in Table 2. And synthesis of the results in Table 3.

**Table 2:** **Overview of Included Studies**

**Author**	**Country**	**Years**	**Type of Study**	**Recruitment period**	**Participants (n)**		**Gender (FM)**		**Age** **(mean ± standard)**	
					**Coil**	**Clip**	**Coil(%)**	**Clip(%)**	**Coil**	**Clip**
**Kelly et al.**	Canada	2010	RCS	1995-2004	778	2342	67	65.5	54.4	53.7
**Choi et al.**	Korea	2016	RCS	2008-2012	8	30	62.5	60	64.75±11.47	53.17±11.96
**Ayling et al.**	Canada	2015	RCS	2005-2006	212	181	NA	NA	NA	NA
**Berro et al.**	France	2019	RCS	2012-2015	48	42	68.8	81	52 ± 10.8	52.6 ± 11.7
**Darsaut et al.**	Canada	2019	RCS	2012-2017	48	55	65	67	56.5	58.5
**Zanaty et al.**	USA	2016	RCS	2010-2015	182	70	73.6	67.1	56.6±12.4	55.9±12.7
**Heit et al.**	USA	2017	RCS	2010-2014	50	50	62	52	55±11.67	50±12.59
**Scheller et al.**	Germany	2018	RCS	2010-2015	45	54	55.8	75.9	60±13.75	57±13.75
**Koh et al.**	Singapore	2013	RCS	2005-2009	23	33	65.2	54.5	52.8 ± 11.6	54.1 ± 13.9
**Shen et al.**	China	2019	RCS	2013-2018	29	65	62	69	65.86±11.597	59.92±10.603
**Zhao et al.**	China	2016	prospective	2010-2012	133	129	46.6	53.5	54.5 **±**11.8	54.4±10.9
**McDonald et al.**	USA	2014	RCS	2006-2011	1227	1227	65	66	53±13.33	53±12.59
**Yu et al.**	China	2007	RCS	1995-2001	80	89	60	62.9	56±13	57±13
**Bekelis et al.**	Lebanon	2016	RCS	2007-2012	2004	1206	73.4	77.2	75.3±6.8	73.5±6.2
**Li et al.**	China	2017	RCS	2002-2010	77	85	59.7	54.1	47.5±10.3	48.1±11.6
**Deutsch et al.**	USA	2018	RCS	2013-2014	15350	6555	65.9	69..0	55.3±33.45	54.1±31.58
**Ryttlefors et al.**	UK	2008	RCS	NA	138	140	68.8	74.3	NA	NA
**Wadd et al.**	Pakistan	2015	RCS	2010-2013	70	70	60	60	52.5±10	51±10
**Hoh et al.**	USA	2010	RCS	2002-2016	3564	5783	68	69	55.0±14.0	53.1±13.0
**Brunken et al.**	Germany	2009	RCS	1990-2004	145	370	NA	NA	53.7±15.5	50.7±16
**Taweesomboonyat et al.**	Thailand	2019	RCS	2002-2018	84	105	81	74.3	64.3±13.9	56.5±11.4
**Zhao et al.**	China	2019	RCS	2008-2015	46	65	52.2	55.4	54.5±11.2	55.5±11.1
**Klompenhouwer et al.**	Netherlands	2011	RCS	2000-2008	230	173	70.4	69.9	53.6	53.1
**Liao et al.**	China	2013	RCS	2008-2009	56	44	68	61	57.91±11.89	56.93±13.75
**Zhang et al.**	China	2012	RCS	2005-2009	76	122	64.5	72.95	51.7±13.0	52.8±10.4
**Lusseveld et al.**	Netherlands	2002	RCS	1983-1999	44	44	66	59	47.0	44.2
**Varelas et al.**	USA	2006	RCS	2000-2004	48	135	45	66	51±15	53±14
**Hoh et al.**	USA	2011	RCS	2002-2007	4306	6593	NA	NA	NA	NA
**Li et al.**	China	2012	RCS	2005-2009	94	92	27.7	32.6	54.7±14.2	53.7±13.8
**Liu et al.**	China	2013	RCS	2001-2005	281	361	60.5	66.8	55.6±15.21	56.90±13.36
**Gross et al.**	USA	2014	RCS	2007-2013	52	203	75	75	NA	NA
**Suzuki et al.**	Japan	2013	Prospective	2006-2007	297	282	65.7	69.9	62.4 ± 14.6	60.2 ± 12.5
**Zaidat et al.**	USA	2009	RCS	1999-2005	98	118	72	72	58 ±1.5	52 ±1.25
**McDougall et al.**	USA	2012	RCT	2003-2007	233	238	71	70	54.3 ± 12.0	53.1 ± 12.8
**Molyneux et al**	Europe	2005	RCT	1994-2002	1073	1070	63	63	52	52
**Koivisto et al.**	Finland	2000	RCT	1995-1997	52	57	46.1	59.6	49±14.25	50±15.25
**Niskanen et al.**	Finland	2004	RCS	1997-2000	68	103	52.9	57.3	54 ±13	54±13
**Rabinstein et al.**	USA	2003	RCS	1990-2000	76	339	62	65	56	53
**Li et al.**	China	2021	RCS	2015-2020	329	329	NA	NA	NA	NA
**Kim et al.**	Korea	2008	RCS	1999-2006	37	35	62.2	57.1	54±13	45±12

Note: NA = not available; RCT = randomized controlled trial; RCS = Retrospective comparative study; FM = female

**Table 3:** Meta-analysis results

**Outcomes**		**Overall effect**			**Heterogeneity**	
		**Effect estimate**	**95% CI**	**p-Value**	**I**^**2**^**(%)**	**p-Value**
**Efficacy**	**mRS(0-2)**	1.327	1.093-1.612	<0.05	38.8	0.091
	**Rebleeding rate**	1.410	1.092-1.822	<0.05	10.6	0.337
	**Mortality**	1.116	1.054-1.180	<0.05	36.9	0.047
	**Complete occlusion**	0.375	0.308-0.456	<0.05	0.0	0.424
**Complications**	**Vasospasm**	0.787	0.649-0.954	<0.05	41.1	0.060
	**Hydrocephaly**	1.143	1.043-1.252	<0.05	30.7	0.173
	**Cerebral infarction**	0.669	0.596-0.751	<0.05	18.9	0.238
	**Neuro deficits **	0.720	0.582-0.892	<0.05	15.6	0.315

Note: mRS = Modified Rankin Scale; GOS = Glasgow Outcome Scale

### Effectiveness of treatment

#### Modified Rankin Scale (MRS)

**Fig. 2:**
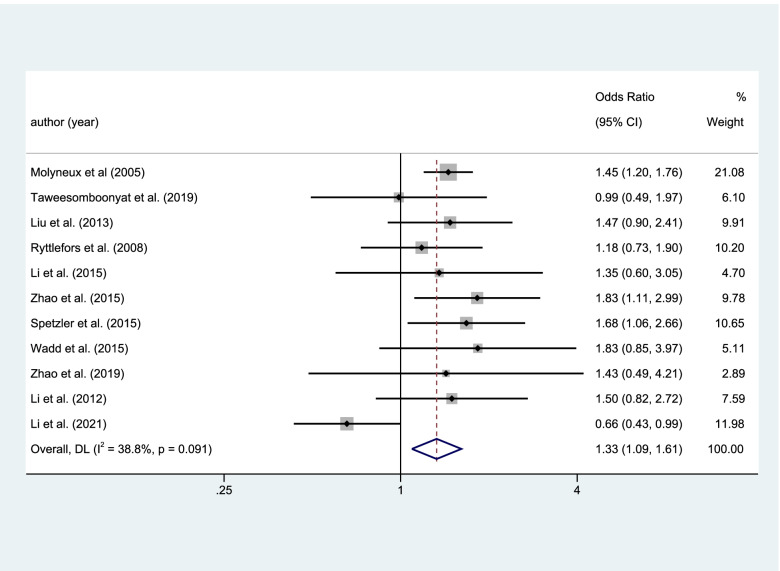
Statistical significance in the results of mRS0-2

The mRS were used in this article to assess the quality of life. There were 11 articles, 4106 patients, including the result of mRS. 65.2% of the patients in the coiling group and 59.9% of patients in the clipping group had a good quality of life defined as mRS0-2. And there was a statistical significance in the results of mRS0-2 (coiling 1523 of 2336 (65.2%) VS clipping1454 of 2428 (59.9%); OR=1.327; CI=1.093–1.612; *p*<0.05; *I*^2^=38.8%; Fig. 2).

#### Rebleeding

**Fig. 3:**
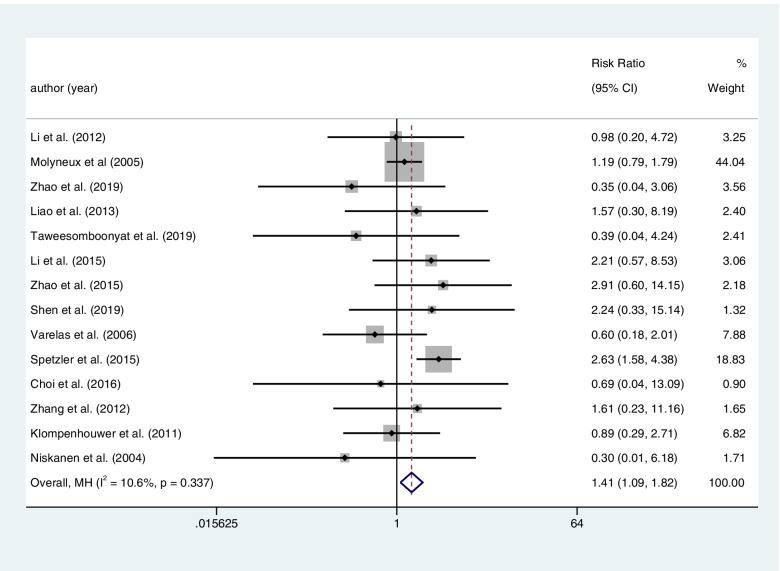
Fourteen articles included a total of 4659 patients with RIAs provided the rate of rebleeding after clipping or coiling. There was higher postoperative rebleeding in the coiling group than the clipping group. And it was associated with a statistical significance

Fourteen articles included a total of 4659 patients with RIAs provided the rate of rebleeding after clipping or coiling. There was higher postoperative rebleeding in the coiling group than in the clipping group. And it was associated with a statistical significance (coiling 128 of 2232 (5.7%) VS clipping103 of 2427 (4.2%); RR=1.410; CI=1.092–1.822; *p*<0.05; *I*^2^=10.6%; Fig. 3).

#### Mortality

**Fig. 4:**
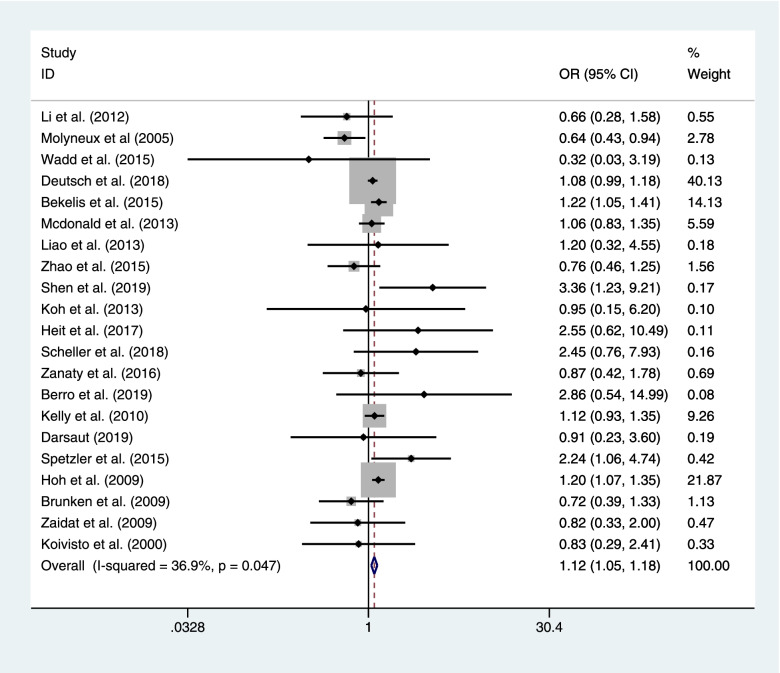
Twenty-one literatures encompassing the rate of mortality after coiling or clipping among 44,909 patients with RIAs

Twenty-one literatures encompassing the rate of mortality after coiling or clipping among 44,909 patients with RIAs. Coiling had a significant effect on the risk of mortality compared with clipping (coiling 3847 of 25,268 (15.2%) VS clipping 2955 of 19,641 (15.0%); OR=1.116; CI=1.054–1.180; *p*<0.05; *I*^2^=36.9%; Fig. 4).

#### Complete occlusion

**Fig. 5:**
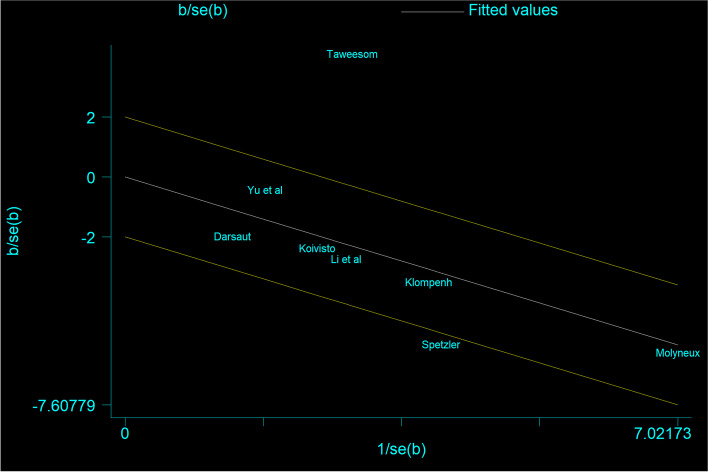
Eight studies included the result of complete occlusion, the result was high heterogeneity

**Fig. 6:**
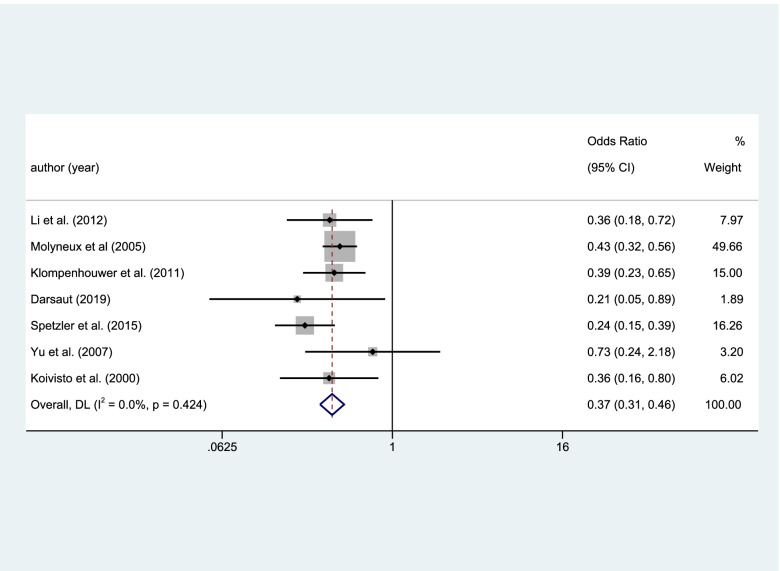
Eight studies included the result of complete occlusion, the result was high heterogeneity

Eight studies included the result of complete occlusion, and the result was high heterogeneity. This study deleted a publication by heterogeneity analysis (Fig. 5). Seven studies included the result of complete occlusion among 2545 patients with RIAs. There was a higher rate of occlusion in the clipping group than the coiling group with a statistical significance (coiling 956 of 1480 (64.6%) VS clipping 881 of 1065 (82.7%); OR=0.375; CI=0.308–0.456; *p*<0.05; *I*^2^=0.0%; Fig. 6).

### Postoperative complications

#### Vasospasm

**Fig. 7:**
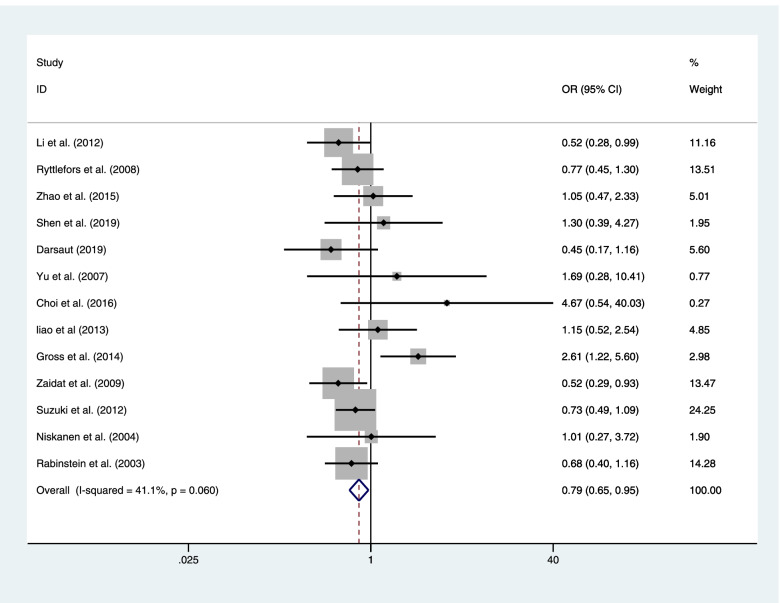
Thirteen publications included a total of 2857 patients with RIAs provided the result of vasospasm after clipping or coiling

Thirteen publications included a total of 2857 patients with RIAs who provided the result of vasospasm after clipping or coiling. There was a less postoperative vasospasm in the coiling group than in the clipping group with a statistical significance (coiling 241 of 1177 (20.5%) VS clipping 416 of 1680 (24.8%); OR=0.787; CI=0.649–0.954; *p*<0.05; *I*^2^=41.1%; Fig. 7).

#### Hydrocephalous

**Fig. 8:**
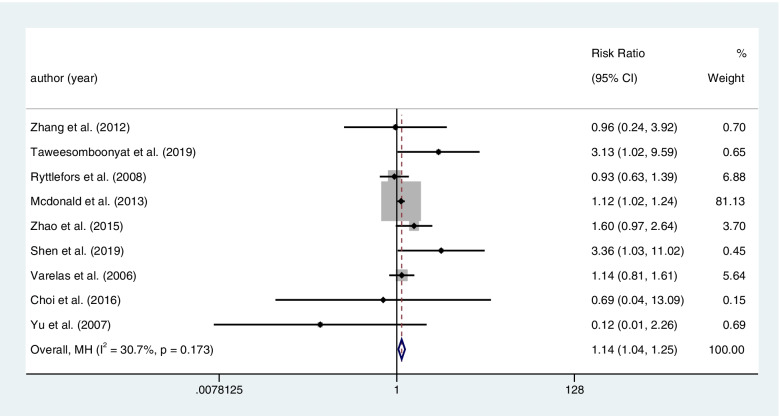
Nine literatures contained the result of hydrocephalous after treatment among 3856 patients with RIAs

Nine literatures contained the result of hydrocephalous after treatment among 3856 patients with RIAs. Coiling had a significant effect on the postoperative hydrocephalous compared with clipping (coiling 611 of 1819 (50.6%) VS clipping 581 of 2037 (39.9%); RR=1.143; CI=1.043–1.252; *p*<0.05; *I*^2^=30.7%; Fig. 8).

#### Cerebral infarction

**Fig. 9:**
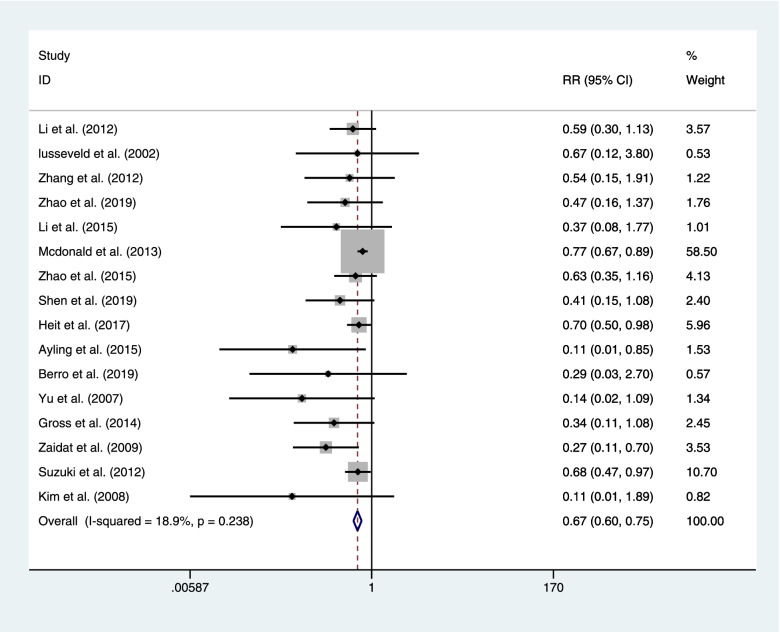
There sixteen articles concluded the result of ischemic infarct after coiling or clipping among 5423 patients

There sixteen articles concluded the result of ischemic infarct after coiling or clipping among 5423 patients. Coiling had a lower postoperative ischemic infarct than clipping with a statistical significance (coiling 375 of 2598 (14.4%) VS clipping 597 of 2825 (21.1%); RR=0.669; CI=0.596–0.751; *p*<0.05; *I*^2^=18.9%; Fig. 9).

#### Postoperative neuro deficits

**Fig. 10:**
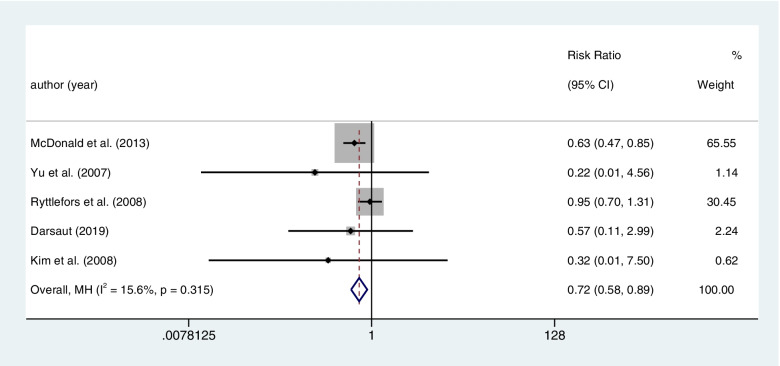
There five articles concluded the result of neuro complications (defined as any new weakness, decreased level of consciousness, paresthesia or cranial nerve deficit), after coiling or clipping among 3076 patients

The five articles concluded the result of neuro complications (defined as any new weakness, decreased level of consciousness, paresthesia, or cranial nerve deficit), after coiling or clipping among 3076 patients. Clipping had a higher rate of postoperative neuro deficits than coiling with a statistical significance (coiling 119 of 1530 (7.8%) VS clipping 167 of 1546 (10.8%); RR=0.720; CI=0.582–0.892; *p*<0.05; *I*^2^=15.6%; Fig. 10).

## Discussion

This meta-analysis summarized the available data with regard to outcomes of patients with RIAs who underwent clipping or coiling procedures systematically. Our meta-analysis included 40 articles involving 60,875 patients with RIAs. And we compared eight outcomes between coiling and clipping including the effectiveness of treatment (mRS, postoperative rebleeding, postoperative mortality, the rate of complete occlusion) and the postoperative complications (vasospasm, hydrocephalous, cerebral infarction, postoperative neuro deficits).

This meta-analysis showed that patients who underwent coiling had a significantly better quality of life (mRS 0-2) than those who underwent clipping at 1 year after treatment. Liu et al. [36] also reported that coiling patients had more good quality of life outcomes than clipping patients at 1 year after treatment. And this result was consistent with ISAT data [5, 7]. Additionally, some articles [23, 28] showed the trend that coiling was related to a higher rate of good outcomes (mRS 0-2) than the clipping group. Yu et al. [21] reported that the result of Glasgow Outcome Score (GOS) (1–3) was lower in endovascular coiling (12/80, 15%) than in microsurgical clipping (30/89, 34%; *p*<0.05). Zhang et al. [19] had the opposite result about the rate of GOS (4–5). Because the admission grade (Hunt-Hess 4–5; *p*<0.01) [27] was associated with poor outcomes, it could explain why there were different results.

250 (23.5%) of 1063 individuals who underwent coiling treatment were dependent or dead at 1 year, compared with 326 (30.9%) of 1055 patients with clipping, an absolute risk reduction of 7.4% (95% CI 3.6–11.2, *p*<0.05) reported by Molyneux et al. [7]. Spetzler et al. [43] also showed coiling was related with a lower rate of mortality. While Shen et al. [18] had the opposite point, their result showed coiling was associated with a higher mortality rate than clipping, this result was similar with our meta-analysis. Our result of mortality was different from published studies, and the difference in categorical data may be one of the reasons [18]. Additionally, this study found that the coiling group has a higher incidence rate of rebleeding rate and a lower complete occlusion rate. It may be associated with higher mortality in the coiling group.

Several articles [18, 19, 31, 32] demonstrated that a trend toward postoperative rebleeding in the coiling group, while other literatures [17, 33] showed the clipping group had a higher rate of rebleeding than the coiling group, and there was no significant difference in their results. In the present article, we find a significantly higher risk of rebleeding in the endovascular coiling group (*p*<0.05). Varelas et al. [33] reported that rebleeding was significantly associated with the ventriculoperitoneal shunt (*p*<0.05), and some published articles suggested that rebleeding also depended on the follow-up period and on the rate of occlusion after endovascular coiling or microsurgical clipping [5, 7] and this meta-analysis also found that clipping was significantly associated with a higher rate of complete occlusion (*p*<0.05), this result was consistent with published studies [28, 31, 35]. Murayama et al. [44] also reported that the rate of complete occlusion was found in 55% of aneurysms, and the lesion neck remnant was identified in 35.4% of aneurysms and the rate of recanalization was up to 20.9%, which was associated with the neck of the aneurysm and size of the dome. And coil compaction and/or loosening and a high rate of the remnant of the neck could also cause recanalization [5, 45].

Our articles showed endovascular coiling was associated with a significantly lower risk of vasospasm, cerebral infarction, post neuro deficits, but with a significantly higher postoperative hydrocephalous than microsurgical clipping.

Li et al. [35, 46] also showed the lower incidence of vasospasm and cerebral infarction in the coiling group. Some other publications [46, 47] were similar to ours about the infarction. One of the vasospasm reasons is that blood degradation products, accumulating in subarachnoid space and reserve as triggers to cause intramural inflammation and endothelial dysfunction [48]. However, there was an argument about vasospasm, someone thought that remove the cisternal blood during clipping would reduce the risk of vasospasm [45]. But this effect could be offset by other effects related with clipping [49], such as surgical operations of the vessels and craniotomy with brain retraction would aggravate the preexisting cerebral vasospasm. And some previous publications suggested that cerebral vasospasm was associated with the incidence of cerebral infarction [50, 51]. There were some other reasons of cerebral infarction: microsurgical clipping blocked some microvascular during surgery, leading to ischemia event. The compression of the small vessels that around the lesion clip may lead to local ischemia [18]. These factors may cause a higher risk of infarction in the clipping group. Additionally, vasospasm-related cerebral infarction significantly influences the rate of mortality following aSAH and cause poor clinical outcomes [52].

The result of postoperative neuro complications was consistent with some published studies [49, 53], and Dumont et al. also analyzed the risk factor of neuro deficits, such as clipping, ventriculostomy, thick clot size, history of hypertension, and intracerebral hemorrhage [49].

So far, some publications had reviewed the morbidity of hydrocephalus after endovascular coiling and microsurgical clipping systematically, while there was no uniform conclusion [21, 54]. While the result of Shen et al. [18] was consistent with this article that coiling was related with a higher risk of hydrocephalous. As is known to all, arachnoid granules absorbed cerebrospinal fluid (CSF), and some CSF was absorbed through the cerebral capillaries. Blood clots may lead to impairment of CSF absorption by disturbing cerebral capillaries and arachnoid villi, causing cerebral hydrocephalous [54]. While clipping could remove the blood clots, improving circulation of CSF, decreasing the risk of hydrocephalous [18]. And the controversy with regard to the result of hydrocephalous may be the different diagnosis criteria of cerebral hydrocephalus [19].

This study has several potential limitations: (1) The included literatures were only 3 RCTs, and this article was limited to the evaluation of short-term results. (2) The sample of some comparative indicators was relatively small.

## Conclusion

Coiling was significantly associated with a better quality of life (mRS0-2), a lower incidence of postoperative complications (vasospasm, cerebral infarction, neuro deficits), and a higher rate of mortality, rebleeding and hydrocephalous than clipping. Additionally, coiling was associated with a lower rate of complete occlusion.

## Supplementary Information

**Additional file 1.** Cochrane Collaboration’s tool for quality assessment RCTs

## Data Availability

The datasets used and analyzed during the current study are available from the corresponding author on reasonable request.

## Abbreviations

RIAs: Ruptured intracranial aneurysms
aSAH: Aneurysmal subarachnoid hemorrhage
SAH: Subarachnoid hemorrhage
ISAT: the International Subarachnoid Aneurysm Trial
IAs: Intracranial aneurysms
RCTs: Randomized controlled trials
mRS: Modified Rankin Scale
GOS: Glasgow outcome score
CSF: Cerebrospinal fluid
PRISMA: Preferred reporting items for systematic reviews and meta-analyses
