# Supplementary material for: Endovascular coiling versus microsurgical clipping for ruptured intracranial aneurysms: a meta-analysis and systematic review
Source: Chin Neurosurg J. 2022 Jul 25;8:17. doi: 10.1186/s41016-022-00283-3 (PMC9310462; doi:10.1186/s41016-022-00283-3)
Supplement: Supplementary file 1 — Additional file 1. Cochrane Collaboration’s tool for quality assessment RCTs [file 41016_2022_283_MOESM1_ESM.docx]

Table 1: Cochrane Collaboration’s tool for quality assessment RCTs.

| **Trials** | **Sequence Generation** | **Allocation Concealment** | **Blinding of Outcome Assessors** | **Adequate Assessment of Outcome** | **Selective Outcome**  **Reporting Avoided** | **Others** |
| --- | --- | --- | --- | --- | --- | --- |
| **McDougall et al.** | Yes | Yes | No | Yes | Yes | Not powered |
| **Molyneux et al.** | Yes | Yes | No | Yes | Yes | Not powered |
| **Koivisto et al.** | Yes | Yes | No | Yes | Yes | Not powered |

**Note: RCTs：Randomized Controlled Trials.**
